# Implications for influenza A virus surveillance in Southeast Asian Region countries: a scoping review of approaches for the surveillance of swine influenza viruses at human-swine interfaces

**DOI:** 10.1136/bmjph-2024-002330

**Published:** 2025-06-18

**Authors:** Subarna Roy, Mohammad Mahmudul Hassan, Gulam Mohd, Abdulkader Suliankatchi Rizwan, Muthappan Sendhilkumar, Jasmine Beryl Lydia, Janana Priya, Manickam Ponnaiah, Pushpa Ranjan Wijesinghe, Edwin Ceniza Salvador, Nilesh Buddha, Ricardo Soares Magalhaes, Manish Kakkar, Manoj Murhekar

**Affiliations:** 1 ICMR - National Institute of Traditional Medicine, New Delhi, New Delhi, India; 2 The University of Queensland School of Veterinary Science, Gatton, Queensland, Australia; 3 ICMR - National Institute of Epidemiology, Chennai, Tamil Nadu, India; 4 Epidemiology, ICMR - National Institute of Epidemiology, Chennai, Tamil Nadu, India; 5 Infectious Hazards Management, WHO Health Emergencies Department, WHO South-East Asian Regional Office, New Delhi, New Delhi, India; 6 WHO Health Emergencies Department, WHO South-East Asian Regional Office, New Delhi, New Delhi, India; 7 Children’s Health and Environment Program, UQ Children’s Health Research Center, The University of Queensland, Saint Lucia, Queensland, Australia

**Keywords:** Zoonoses, Risk Assessment, Public Health, Preventive Medicine, Epidemiologic Factors

## Abstract

**Background and objectives:**

The Southeast Asian Region (SEAR) faces a heightened risk of reassortment of circulating influenza viruses in swine populations due to the dense cohabitation of humans, poultry and swine. However, there is currently no specific guidance on conducting influenza surveillance at the human-swine-poultry interfaces within SEAR. This study conducted a scoping review to characterise the objectives and frameworks of influenza A Virus (IAV) surveillance systems established at human-swine exposure sites globally.

**Design:**

A literature search was performed for studies published until June 2024 that reported on IAV survey and surveillance activities at swine farms, slaughterhouses, agricultural fairs, animal markets, backyard farms and among swine workers.

**Data sources and extraction:**

Data were extracted from relevant articles using PubMed, Scopus and Web of Science, following Preferred Reporting Items for Systematic Reviews and Meta-Analyses guidelines for scoping reviews. Extracted data were organised in MS Excel, with analyses stratified by IAV surveillance objectives.

**Results:**

Of 42 studies meeting inclusion criteria, most were short-term and project-based. Half of the studies (50%; 21/42) were conducted in Asia, strongly focusing on virological monitoring as the main surveillance objective (69%; 29/42). Swine farms were the primary setting for surveillance (61.90%; 26/42), with active surveillance employed in most cases (90.48%; 38/42). Sampling techniques included nasal, tracheal and faecal swabs, along with serum and lung tissue, with most sampling targeting swine (73.81%). Other targets included swine and humans (11.90%); swine, humans and environmental samples (7.14%); humans only (4.76%); and swine and poultry (2.38%). Testing relied heavily on PCR and whole-genome sequencing, used by half of the studies (50%; 21/42) alongside RT-PCR and ELISA for detecting IAVs.

**Conclusion:**

This study reveals considerable variability in IAV survey and surveillance across human-swine-poultry interfaces. Establishing standardised, objective-based protocols for such surveillance is crucial to strengthening IAV’s global preparedness and response capabilities and benchmarking progress towards zoonotic risk reduction.

**WHAT IS ALREADY KNOWN ON THIS TOPIC**

- Swine serve as mixing vessels for influenza viruses, posing a risk of reassortment and zoonotic spillover.
- Existing influenza A virus (IAV) surveillance is fragmented, predominantly short-term, and lacks standardised protocols.
- There is no specific guidance for conducting IAV surveillance at human-swine-poultry interfaces, especially in the Southeast Asian Region (SEAR).

**WHAT THIS STUDY ADDS**

- This study identifies significant variability in IAVs surveillance, with most efforts being short-term and virology-focused.
- It highlights the dominance of active surveillance at swine farms and the reliance on PCR and genomic sequencing for virus detection.
- The findings emphasise the need for standardised, objective-based surveillance frameworks to enhance global preparedness and zoonotic risk mitigation.

**HOW THIS STUDY MIGHT AFFECT RESEARCH, PRACTICE OR POLICY**

- This study underscores the need for standardised, objective-driven surveillance protocols to improve the consistency and effectiveness of IAV monitoring.
- It can guide future research in developing more comprehensive surveillance strategies that integrate human, swine and environmental components.
- Policymakers can use these insights to strengthen zoonotic disease preparedness and establish region-specific surveillance frameworks, particularly in SEAR.

Policymakers can use these insights to strengthen zoonotic disease preparedness and establish region-specific surveillance frameworks, particularly in SEAR.

## Introduction

 Influenza viruses, especially influenza A viruses (IAVs), are of significant public health importance due to their potential for evolving into pandemic strains.^1^ Influenza viruses also circulate in swine populations, and pigs are considered the mixing vessels for zoonotic influenza viruses in that novel viruses can reassort in swine populations as a result of reverse zoonotic transmission from humans and cross-species transmission with avian strains.^2^,^4^

The swine-derived H1N1 strain caused a global pandemic in 2009, first detected in Central West Mexico.^5 6^ A novel variant H3N2 virus (H3N2v) containing the M segment of the H1N1pdm was associated with a zoonotic transmission event in 2011.^7^ To date, a total of 429 H3N2v and other swine H1N1 and H1N2-linked zoonotic infections have been reported.^8^ Numerous new genotypes of IAV circulating in swine with new combinations continue to be reported in swine populations in China and Europe, underlining the continuing threat these viruses can pose to global public health.^9^,^11^

The spillover of zoonotic influenza viruses to humans, including IAVs, depends on the interplay of several factors, such as disease dynamics in the reservoir host and the context of exposure to the pathogen.^12 13^ Generally, people who work in pig farming,^14^,^16^ the pork industry^17^ and veterinarians^18^,^21^ are considered to be at higher risk of infection. The risk of IAV spillover is enhanced in intensive swine farming and trade, swine exhibitions and agricultural fairs,^22^,^24^ slaughterhouses^14 20^ and live animal markets.^15 25 26^ Additional interfaces of exposure to IAVs include low biosecurity backyard swine farming systems, and wild boar movements towards human settlements are also interfaces of exposure for IAVs.^22 27^ Swine IAVs have been found to be associated with important environmental factors such as seasonal variation in temperature, with lower odds of circulation attributed to summer months compared with cooler seasons.^28^,^30^ A higher prevalence of IAVs circulating in swine has also been reported to be associated with farm-level biosecurity indicators such as the total number of pigs on site, presence of other animal species in farms,^31^ uncontrolled farm access and open partitions between pens in fattening units.^32^

Due to its combined high density of human and pig populations, countries in the Southeast Asian Region (SEAR) are considered to be at greater risk of the emergence of new pandemic-prone IAVs.^33^,^35^ Commonly, swine husbandry systems in SEAR countries include backyard,^36 37^ mixed or integrated farming, large scale or commercial pig farming,^38^ and live animal markets.^39^,^42^ These demographic and husbandry characteristics enhance SEAR’s vulnerability to circulating zoonotic influenza viruses, particularly the current H5N1 which has recently gained increased cross-species pathogenicity. A number of H5N1 outbreaks are being reported in SEAR countries such as Cambodia and Vietnam, which have confirmed human and poultry cases with no swine cases reported to date.^43^ Current H5N1 circulating widely in dairy cattle in the USA has recently been reported in goats.^43 44^ If this strain adapts to swine populations in SEAR counties due to the high-risk demographic and husbandry characteristics in the countries, there is a significant risk of a reassortment event, highlighting the broader potential for cross-species transmission and global spread, which can lead to effective human-to-human transmission. Given the central role of swine populations for influenza reassortments leading to potential pandemic strains, there is a need to look at the evidence on protocols to effectively deploy influenza surveillance in human-swine-poultry interfaces in SEAR, so countries can detect emerging strains of concern.

The countries’ preparedness for the emergence of novel zoonotic IAVs from swine populations includes the implementation of robust surveillance systems that are objective-based, locally appropriate, cost-effective and sustainable. The Food and Agriculture Organization (FAO), the World Organisation for Animal Health (WOAH) and various national veterinary authorities in developed nations have put together valuable guidelines and surveillance protocols for IAVs circulating in swine and other species.^45^ These guidelines provide high-level guidance on monitoring influenza viruses in animal populations to detect potential threats to human health and to prevent the spread of novel strains. The recent incursion of the H5 virus in backyard swine production in the USA highlights the critical need for robust swine influenza surveillance to detect emerging threats early, assess zoonotic risks and prevent potential spillover events.^46^ Given the high cross-sectoral risks of zoonotic influenza spill-over in SEAR countries, preparedness and response structures in those countries would benefit from surveillance systems designed to detect the spill-over of zoonotic influenza from poultry to swine and from poultry/swine to humans. To support local decisions for cross-sectoral swine influenza surveillance design, there is a need to review the existing literature for globally documented surveillance experiences for IAVs at the human-swine interfaces with relevance to SEAR countries globally. The lack of fit-for-purpose guidance for the context of spill-over detection across livestock species and between livestock and humans in animal interface settings poses a significant limitation to preparing SEAR countries to prevent future influenza pandemics.

In this study, we aimed to review the characteristics of established IAV surveillance systems and describe the attributes of such systems in terms of types of their objectives, interfaces and risk groups targeted, sampling strategies and virus detection methods.

## Methods

### Search strategy

We conducted a scoping review of literature following the reporting guidelines of the Preferred Reporting Items for Systematic Reviews and Meta-Analysis extension for Scoping Reviews (PRISMA-ScR) and the PRISMA. The guiding research question for the scoping review was ‘What are the design characteristics of global swine influenza surveillance efforts in human and animal populations?’. Our search strategy was guided by terms referring to the Population, Exposure, Comparator, and Outcome used in the PRISMA guidelines. We used the following terms in our search strategy: population terms (“Swine population” OR “Human population”, Exposure terms: “Surveillance” OR “Survey”, Comparator terms – “swine farm” OR “slaughterhouse” OR “animal markets” OR “agriculture fairs” OR “backyard production”) and outcome terms (“Influenza virus”). Three databases (PubMed, Scopus and Web of Science) were searched from their inception to July 2024. We discussed this with key stakeholders such as veterinary researchers, public health researchers, and epidemiologists, and we used the published literature to list relevant keywords to search and retrieve relevant articles. For advanced search strategies, we used [Title/Abstract] in PubMed, [Title OR Abstract] in Scopus and [Title OR Abstract] in Web of Science. A comprehensive overview of the search strategies, including the databases used, keywords and search parameters, is provided in online supplemental table S1. All records (n=1993) were imported into EndNote X20 (Clarivate Analytics, Philadelphia, PA, USA) to remove duplicate entries (n=373).

### Eligibility criteria

Retrieved articles were checked for duplicates and non-relevance. After removing duplicate articles, the titles and abstracts of articles were screened by three independent readers (M.M.H., J.P. and J.B.L) to identify the relevant articles and used the inclusion and exclusion criteria to include the articles. Initial duplicates were removed using EndNote, and the remaining articles were transferred to the Rayyan system (https://rayyan.qcri.org/), where further screening was conducted based on inclusion and exclusion criteria. All three readers independently reviewed the articles, and if any conflict arose, it was discussed, thoroughly re-evaluated and resolved through consensus. The inclusion criteria were original research articles, published in English, detailing surveillance objectives and survey/surveillance types (eg, active, passive, joint surveillance). Articles excluded included reviews, editorials, or book chapters and were not published in English (other language). No restriction on the publication date was imposed to cover all the prior information on the surveillance of swine influenza globally.

**Figure 1:**
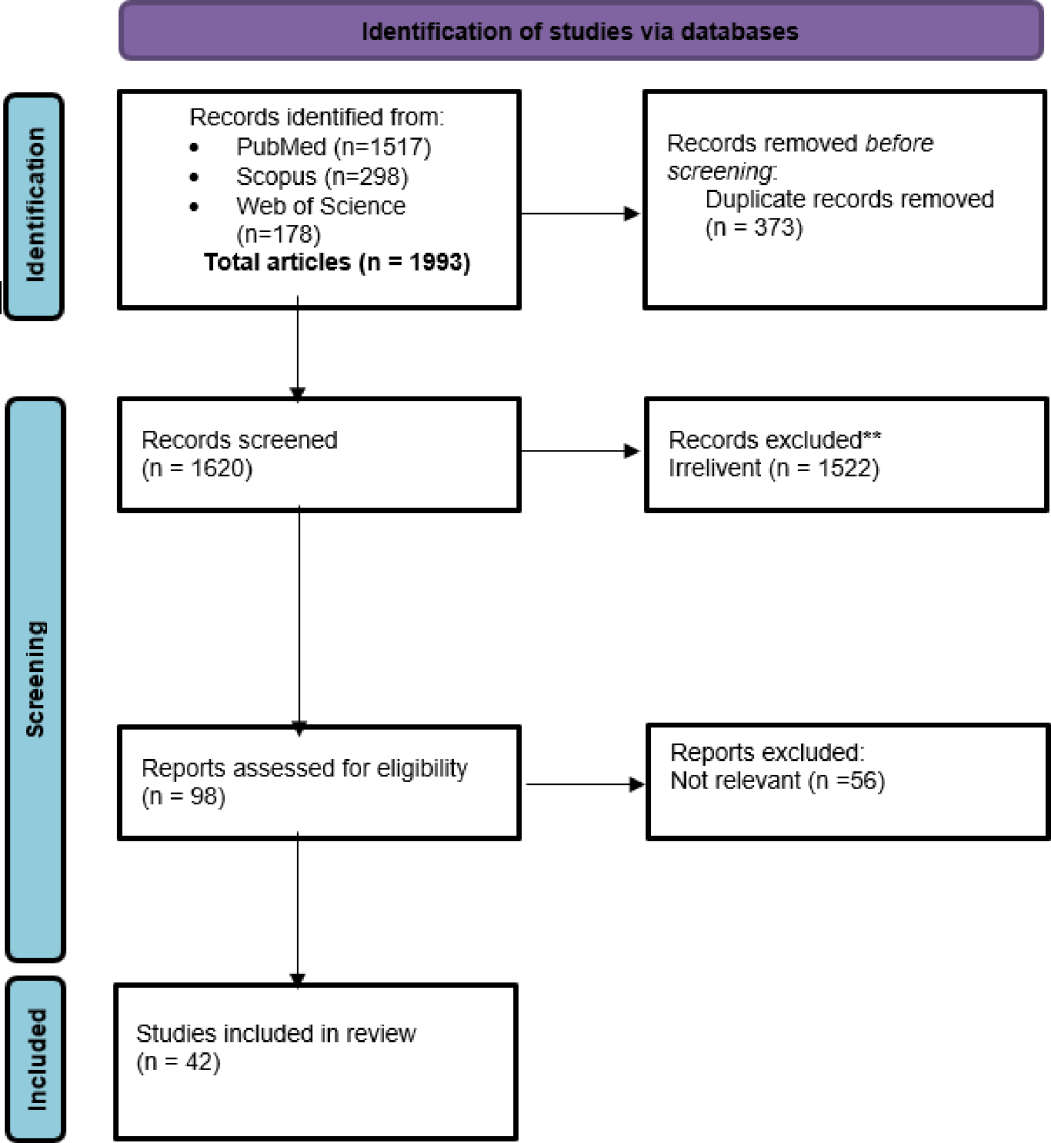
Flow chart demonstrating the scoping review process of this study.

After title/abstract and full-text screening, 42 relevant articles were selected for further analysis, and data on potential swine influenza surveillance were extracted (figure 1).

### Data extraction

The details of relevant information extracted from selected articles were stored in a data extraction tool developed in MS Excel and are summarised in online supplemental table S2. In brief, data extracted from each article focused on the design characteristics of surveillance established for swine influenza at human-swine interfaces, including objectives of the surveillance (ie, objective 1 (monitor the intensity, geographical spread and temporal patterns of swine influenza); objective 2 (monitor severity and risk factors for severe disease and assess the impact on healthcare and/or animal production systems of swine influenza); objective 3 (monitor changes and characteristics of circulating and emerging virological changes of swine influenza viruses to inform the development of medical countermeasures); and objective 4 (assess vaccine effectiveness against swine influenza disease)), sampling setting (ie, swine farms, slaughterhouses, animal markets, agricultural fairs, backyard production systems and swine workers), target sampling (swine, humans and environment), type of survey/surveillance (active, passive and joint survey/surveillance), period/duration of the surveillance (months/years of the surveillance), country (Asia, Europe, North America, Africa and Russia), sampling strategy (random, clinical signs, serial sampling and convenience), case definition, type of samples collected (nasal swabs, tracheal swabs, faecal swabs, serum and lung tissue), frequency of sample collection (daily, weekly once, monthly once), tests performed (ELISA, PCR and whole-genome sequencing) and type of virus (influenza A and B virus, H1N1, H1N2, H3N1, H3N2 and mixed). Some studies reported having multiple surveillance objectives, and this was recorded in the data extraction tool. For the purpose of our study, active surveillance was defined as proactive data collection through targeted sampling, testing and investigations to detect diseases, providing timely and accurate insights. In contrast, passive surveillance relies on routine reports from veterinarians, farmers or healthcare providers, making it cost-effective but potentially prone to underreporting or delays in detection. For Objective 2, risk factors and severity indicators were analysed, including morbidity and mortality rates.

### Data analysis

**Figure 2:**
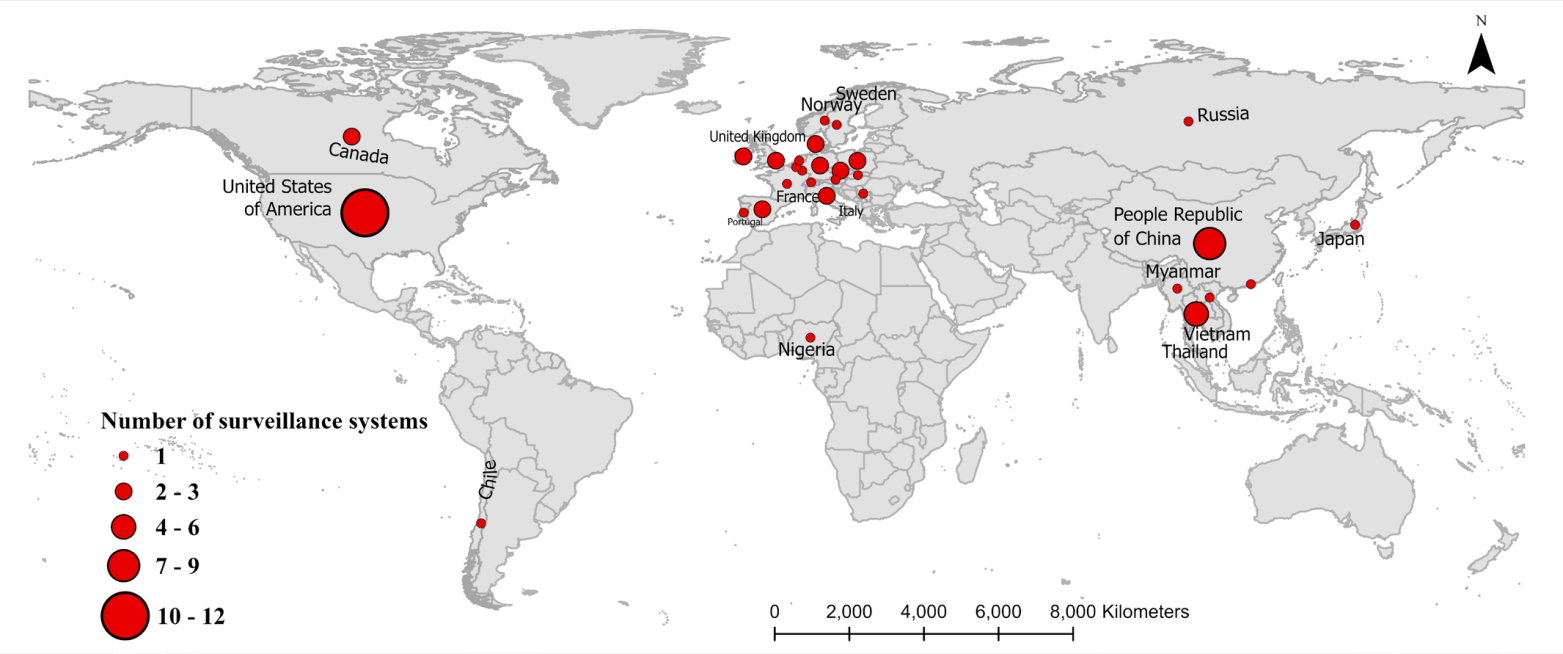
Global distribution of influenza A virus’ (IAV) surveillance systems at the human-animal interface.

For data analysis, we have analysed the extracted information based on (a) general characteristics of studies included in the review and then (b) based on each of the four surveillance objectives. The distribution of the number of IAV surveillance systems at the human-animal interface was visualised using ArcMap (V.10.8) in the map (figure 2).

### Protocol registration

The protocol for this scoping review has been registered with PROSPERO with record number CRD 42022297929.

## Results

### Screened and included studies

We retrieved a total of 1993 articles, from PubMed (n=1517), Scopus (n=298) and Web of Science (n=178) articles, respectively (figure 1). Duplicate articles were removed, and a total of 1620 articles were considered for the initial title and abstract screening. We screened the title and abstract of the articles and finalised 98 articles for full-text screening. We excluded 56 articles during the full-text review as they were not directly related, that is, reviews, editorials or book chapters, and not published in English (other language). This final study selection step yielded a total of 42 articles for full in-text data extraction (figure 1).

### General characteristics of selected articles

#### Types, settings and locations of surveillance

The majority of selected studies were performed as standalone surveys as part of cross-sectional research projects (n=37/42; 88.10%). The majority (38/42; 90.48%) of studies reported active surveillance approaches, followed by passive (3/42; 7.14%) and joint surveillance (1/42; 2.38%). In terms of surveillance settings, most of the surveillance studies were done in swine farms (26/42; 61.90%). The other surveillance setting included a combination of swine farms and slaughterhouses (5/42; 11.90%), slaughterhouses alone (4/42; 9.52%), animal markets (3/42; 7.14%), agricultural fairs (3/42; 7.14%) and backyard farms (1/42; 2.38). Half of the studies were conducted in Asia (21/42; 50%), followed by North America (13/42; 30.95%), Europe (5/42; 11.90%), and Africa and Russia combined (2/42; 4.76%) (figure 2). The majority of studies were conducted after the year 2000 (28/42; 66.67%); however, the first published reported IAV surveillance based on our scoping review was in 2008.

#### Sampling and laboratory techniques of surveillance

Most of the targeted sampling focused on swine (31/42; 73.81%), with additional targets being swine and human (5/42; 11.90%), swine, human, and environment (3/42; 7.14%), human only (2/42; 4.76%), and swine and poultry (1/42; 2.38%). The total number of samples taken within identified surveillance studies varied between 16 and 36 417. Most studies collected nasal swabs alone (19/42; 45.52%) followed by a combination of nasal swabs, tracheal swabs and serum (11/42; 26.19%), combinations of nasal swabs, tracheal swabs and aerosol (7/42; 16.67%), serum alone (3/42; 7.14%) and lung tissues (2/42; 4.76%) (table 1) as a sample type for testing. The most common laboratory testing methods were a combination of PCR and whole-genome sequencing (21/42; 50%) followed by PCR (12/42; 28.50%) alone, whole-genome sequencing (4/42; 9.52%), ELISA (4/42; 9.52%), and a combination of ELISA and whole-genome sequencing (1/42; 2.38%) (table 1).

**Table 1:** Summary characteristics of the surveillance system related to swine influenza globally

Characteristics (N=42)	Variables	n; %
Types of surveillance	Active surveillance	38; 90.48%
	Passive surveillance	3; 7.14%
	Joined surveillance	1; 2.38%
Surveillance setting	Swine farms	26; 61.90%
	Swine farms and slaughterhouses	5; 11.90%
	Slaughterhouses	4; 9.52%
	Animal markets	3; 7.14%
	Agriculture fair	3; 7.14%
	Backyard production system	1; 2.38%
Species sampled	Swine	31; 73.81%
	Swine and human	5; 11.90%
	Swine, human and environment	3; 7.14%
	Human	2; 4.76%
	Swine and poultry	1; 2.38%
Country of surveillance	Asia	21; 50.00%
	Europe	5; 11.90%
	North America	13; 30.95%
	South America	1; 2.38%
	Africa	1; 2.38%
	Russia	1; 2.38%
Sample type used for surveillance	Nasal swabs	19; 45.24%
	Nasal and tracheal swabs, serum samples	11; 26.19%
	Nasal swabs, faecal and aerosol samples	7; 16.67%
	Serum samples	3; 7.14%
	Lung tissue samples	2; 4.76%
Laboratory techniques used for surveillance	PCR and whole-genome sequencing	21; 50.00%
	PCR	12; 28.57%
	Whole-genome sequencing	4; 9.52%
	ELISA (antibodies)	4; 9.52%
	ELISA (antibodies) and whole-genome sequencing	1; 2.38%

#### Objectives of surveillance system

The highest proportion of reviewed articles (29/42; 69.05%) focused on ‘objective 3’ (ie, detect virological changes) of the swine influenza surveillance followed by ‘objective 1’ related to monitoring spatial and temporal patterns of swine influenza incidence (26/42; 61.90%) (table 2). One-third of reviewed articles (14/42; 33.33%) primarily focused on describing ‘objective 2’ related to monitoring severity, and risk factors for severe disease, and assessing the impact on healthcare systems of swine influenza, and none of the reviewed articles addressed objective 4.

**Table 2:** Summary objectives of the surveillance system related to swine influenza globally

Objectives (N=42)	n; %	Main type of surveillance	Main type of settings	Main target host	Main type of samples taken	Main laboratory tests used
Objective 1: Monitor the intensity, geographical spread and temporal patterns of swine influenza	26; 61.90%	Active	Swine farms	Swine	Nasal swabs	PCR followed by whole genome sequencing
Objective 2: Monitor severity and risk factors for severe disease and assess the impact on healthcare and or animal production systems of swine influenza	14; 33.33%	Active	Swine farms	Swine	Nasal swabs	PCR
Objective 3: Monitor changes and characteristics of circulating and emerging virological changes of swine influenza viruses to inform the development of medical countermeasures	29; 69.05%	Active	Swine farms	Swine	Nasal swabs	PCR followed by whole genome sequencing

##### Characteristics of surveillance systems for ‘objective 1’

###### Type of surveillance approach

Our results show that surveillance systems aimed at objective 1 primarily employ active (22/26; 84.62%) followed by passive surveillance (3/26; 11.54%) systems. However, the review also identified the emergence of joint surveillance systems (1/26; 3.84%) within swine production, which integrate both active and passive approaches to enhance disease detection and control through collaborative efforts across various stakeholders and jurisdictions.

###### Type of settings targeted

Among the identified settings, swine farms constituted the majority (17/26; 65.38%), followed by a combination of swine farms and slaughterhouses at 13.38% (4/26). Additionally, surveillance was conducted in other settings including slaughterhouses alone (3/26; 11.54%), animal markets (1/26; 3.84%), agricultural fairs (3/26; 11.54%) and backyard production systems (1/26; 3.84%), highlighting the diversity of environments targeted for monitoring.

###### Type of sample targeted

Swine was the predominant sampling target (19/26; 73.08%), followed by swine and human (3/26; 11.54%); swine, human and environment (2/26; 7.69%); swine and poultry (1/26; 3.84%); and human only (1/26; 3.84%). Nasal swabs were identified as the predominant sample type in isolation alone (10/26; 38.46%) and combined with others, comprising 84.61% (22/26) of the sampled materials. Following nasal swabs, serum alone and combined with other swab samples (10/26; 38.46%), and lung tissues combined with other swab samples (3/26; 11.54%).

###### Type of laboratory techniques used

In objective 1, PCR and whole-genome sequencing combined (11/26; 42.31%) were the predominant techniques used for swine influenza surveillance, followed by PCR, ELISA and whole-genome sequencing of the reported methodologies. PCR (9/26; 34.62%) was frequently employed as the initial diagnostic tool, followed by whole-genome sequencing for in-depth genetic analysis. Additionally, ELISA (4/26; 13.38%) and whole-genome sequencing alone (1/26; 3.84%) were used as the primary technique.

### Characteristics of surveillance systems for ‘objective 2’

#### Type of surveillance approach

Within ‘objective 2’, the majority (12/14; 85.71%) of articles recommended the implementation of active surveillance methods to monitor the severity of swine influenza and identify pertinent risk factors. Key indicators of disease severity, including morbidity and mortality rates, were reported as percentages to provide a clearer understanding of outbreak impact. These findings highlight the importance of continuous monitoring and targeted interventions to mitigate potential risks of IAV transmission at the human-animal interface.

#### Type of settings targeted

‘Objective 2’ was observed across diverse settings such as swine farms (10/14; 71.43%), slaughterhouses (2/14; 14.29%) and animal markets (2/14; 14.29%).

#### Type of sample targeted

Swine was the most sampling target (10/14; 71.43%), followed by swine, human and environment (2/14; 14.29%); swine and human (1/14; 7.14%); and human only (1/14; 7.14%). Nasal swabs predominated taken from healthy animals/people were the most frequently used sample type in isolation alone (7/14; 50%) and combined with other swabs, comprising 100% (14/14) of the sampled materials. Following nasal swabs, serum was combined with other swab samples (3/14; 21.43%), and faecal and aerosol samples were combined with other swab samples (3/14; 21.43%).

#### Type of laboratory techniques used

In objective 2, PCR (8/14; 57.14%) was the predominant technique used for swine influenza surveillance, followed by a combination of PCR and whole-genome sequencing (5/14; 35.71%) and in a combination of ELISA and whole-genome sequencing (1/14; 7.14%) of the reported methodologies. The PCR was frequently employed as the initial diagnostic tool, followed by whole-genome sequencing for in-depth genetic analysis.

### Characteristics of surveillance systems for ‘objective 3’

#### Type of surveillance approach

For manuscripts reporting Objective three as their main surveillance objective, active surveillance (25/29; 86.21%) emerges as the cornerstone to achieve this goal, playing a pivotal role in monitoring virological changes and informing intervention strategies.

#### Type of settings targeted

Swine farms (20/29; 68.96%) along with slaughterhouse were identified as the predominant surveillance setting, indicating the critical importance of monitoring viral dynamics at the primary source of swine influenza transmission. However, among the identified settings, swine farms constituted the majority (17/29; 58.62%), followed by a combination of swine farms and slaughterhouses at 20.69% (6/29). Additionally, surveillance was conducted in other settings including slaughterhouses alone (3/29; 10.34%), agricultural fairs (3/29; 10.34%), animal markets (1/29; 3.45%) and backyard production systems (1/29; 3.45%) used in ‘objective 3’.

#### Type of sample targeted

Swine was the predominant sampling target (23/29; 79.31%), followed by swine and human (4/29; 13.79%); swine, human and environment (1/29; 3.45%); and swine and poultry (1/29; 3.45%). Nasal swabs from healthy animals emerged as the most commonly identified sample type alone and combined with others, comprising 86.21% (25/29) of the sampled materials. Following nasal swabs, other types of swabs, lung tissues alone and combined with other samples (13/29; 44.83%), and serum combined with other samples (7/29; 24.14%) were also frequently collected for surveillance purposes. This diversity in sample types underscores the multifaceted approach taken to capture various aspects of swine influenza epidemiology in the context of objective 3.

#### Type of laboratory techniques used

In objective 3, PCR and whole-genome sequencing combined (18/29; 62.07%) were the predominant techniques used for swine influenza surveillance, followed by PCR, whole-genome sequencing and ELISA of the reported methodologies. PCR (6/29; 20.69%) was frequently employed as the initial diagnostic tool, followed by whole-genome sequencing for in-depth genetic analysis. Additionally, whole-genome sequencing alone (4/29; 13.79%) was used as the primary technique, highlighting its importance in directly characterising viral genomes without prior PCR amplification.

## Discussion

Current global circulation and multispecies outbreaks of AIV/H5N1 pose significant public health concerns, particularly if this strain gains access to swine populations. Enhanced swine production biosecurity coupled with objective-oriented, sustained swine IAV surveillance is paramount to limit poultry-swine spillover and monitor the circulation and/or exposure to AIV/H5N1 within this critical population. With that in mind, the FAO and WOAH have released guidelines to support surveillance for IAVs in animal populations, with a particular focus on the poultry value chain where animal health risks are considered to be highest. From a public health perspective, spillover from poultry to a mammal bridging species is of concern due to virological adaptations and mutations that can result from infection, and swine are traditionally considered a high-risk species for IAV spillover to humans. In the context of SEAR countries, interfaces of exposure that include humans, swine and poultry are particularly prevalent and constitute foci of risk for the emergence of novel IAVs of pandemic potential. However, the lack of specificity in current surveillance guidance for those settings poses a significant gap in SEAR countries’ preparedness and response.

Beyond the exception of a few robust examples of cross-sectoral swine IAV surveillance, this review documented that the vast majority of swine-focused IAV surveillance systems reported in the literature were project based and short lived. We identified three passive surveillance systems^47^,^49^ involving sampling primarily from swine pig farms. These systems were crucial in monitoring disease dynamics in swine populations, providing valuable insights into potential spillover risks and emerging threats. The Réseau national de Surveillance des Virus Influenza A chez le Porc (RESAVIP) surveillance network, an event-based passive surveillance system in France between 2011 and 2018, was established to understand the diversity and dynamics of IAVs circulating in pigs.^50^ The veterinarian either collected samples after a report of an outbreak from a farmer or during monthly herd visits.^48^ An in-depth passive surveillance conducted among 2500 European swine holdings from April 2015 to January 2018 found 31 distinct genotypes of swine IAV, the virulence consequences of which were unknown.^49^ Another 8-year passive surveillance study carried out in Denmark, where samples were received every month from individual pigs with acute respiratory diseases during the surveillance period, showed a higher detection of IAVs during the autumn and winter months.^47^ Moreover, the major four lineages of swIAV (H1avN1av, H1huN2, H1pdmN1pdm and H3N2) were encountered in the majority of the farms surveyed through this system.^47^ European Surveillance Network for Influenza in Pigs (ESNIP) is an extensive swine IAV surveillance programme conducted in three phases with the primary objective of enhancing the epidemiological understanding of IAV circulating in swine in European countries to standardise diagnostic techniques.^51 52^ ESNIP was implemented in three phases: the first phase (ESNIP1) during 2001–2004 in ten European countries; the second, ESNIP2, during 2006–2009;^53^ and the third, ESNIP3, during 2010–2013.^54^ ESNIP-3, which predominantly followed a passive surveillance system, conducted extensive virological monitoring across 17 European countries involving more than 9000 herds.^55^ The methodology of this programme was divergent and adapted according to the needs of each country.^54^ However, setting up passive or abattoir-based surveillance was proven to be cost-effective and sustainable and, therefore, can be adapted to SEAR countries. This finding can partly be explained by the fact that the swine IAV is currently not a notifiable disease by the WOAH, and thereby, surveillance in this population is not warranted.^56^ For example, studies from Hong Kong, Southern China and the USA reporting abattoir and animal fair surveillance in swine ranged between 3 and 12 years follow-up until they were discontinued.^57^,^59^ The lack of sustained swine IAV surveillance in SEAR countries poses a significant risk to the swine production sector as well as to consumers. Indeed, our study has shown that fewer of the examples in the literature were of swine IAV surveillance coupled with human surveillance.^60 61^ Anticipation and rapid recognition of spillover events in SEAR countries can be provided by surveillance designs that bring sympatric data from swine and humans together for virological and exposure trend analysis. For example, focusing on both swine and human species, a 2-year prospective cohort study was conducted at a single pork production company in Iowa.^62^ The study sampled approximately 200 individuals working in the company, drawing blood samples two times during the study (fall and spring, a few a third time after vaccination) and reporting influenza-like illness (ILI) symptoms. This study was able to capture about 20 swine herd ILI outbreaks.^63^ Our scoping review has shown that there is a significant gap in surveillance designs in that only 19% of studies combined animal sampling with some form of human sampling.

The lack of sustained surveillance efforts to swine IAV surveillance limits the ability to monitor the evolution of new genotypes adequately, the emergence of strains with enhanced zoonotic potential and the understanding of diversity and dynamics of IAVs not only among pigs but also at human-pig-poultry interfaces which are common in SEAR countries. To monitor the antigenic and genomic properties of IAVs in the USA, pigs were sampled at 53 agricultural fairs over a period of 3 years. At the end of each swine exhibition, pigs were visually examined, and nasal swabs were collected irrespective of the swine health status to determine the circulation of IAV in swine at the fairs to protect swine and human health. While for developing countries, swine IAV surveillance at agricultural fairs may yield better sensitivity for detection, in SEAR countries, the risk of transmission is considered higher in the farming sector, given the diversity and lower level of biosecurity standards. Our results suggest that most swine surveillance reported in the literature was found to focus on commercial pig farms, with only one report on the backyard production sector.^3047^,^49 54 60 61 64^ The lower level of biosecurity afforded by backyard swine production places this sector as a high-risk interface for AIV spillover, which should be under increased scrutiny. The low mortality, high morbidity and self-limiting nature of these viruses in swine populations make them difficult to detect clinically in commercial farms.^82^ One alternative adaptable to SEAR countries would be for surveillance to be focused on smallholder farms and abattoir-based sentinel sites, as exemplified in the studies we reviewed from Vietnam^79 83^ and China.^84^

When designing a surveillance system, the first consideration is the surveillance objective. Our results have shown that from the surveillance studies, none had been set out with the objective of assessing vaccine effectiveness against swine influenza infections (ie, objective 4). Our results demonstrated that more than two-thirds of the studies were designed with the objective of detecting virological changes in IAVs (ie, ‘objective 3’). Studies that aimed to address objective 3 focused primarily on swine sampling, and very few (17%) combined swine and human sampling or any other species, such as poultry. The lack of cross-species data from sympatric settings limits data insights for action. Furthermore, we have noted that none of the studies provided a rationale for the choice of the sampling frame. This information is quite important to be clearly specified, particularly for a surveillance objective that aims to detect a spillover event. To maximise the probability of detection, the virological surveillance needs to be risk-based whereby the probability of detection is influenced by the proximity to potential spillover species such as poultry and backyard swine systems. In the context of SEAR countries, consideration of risk-based surveillance designs is paramount for the selection of the sampling sites or populations within the sites, given the sympatric nature of exposure interfaces.

A second group of studies had monitoring spatial and temporal patterns of swine influenza incidence as their main objective (‘objective 1’). To achieve this objective, surveillance designs need to be implemented as active surveillance in order to have robust measures of infection exposure, given that most infections are asymptomatic. This active surveillance approach enables real-time data collection, facilitating a more comprehensive understanding of the disease dynamics and aiding in the development of targeted intervention strategies. Such proactive measures are crucial for mitigating the potential impact of swine influenza outbreaks on both animal and public health. Furthermore, surveillance systems aiming at objective 1 would be more sensitive to target the sampling at markets/slaughterhouses and particularly the environmental sampling to monitor trends in circulating viruses. It is interesting that most reviewed studies that aimed to look at objective 1 have focused their sampling in the farm setting, which is prone to the effect of cleaning operations and non-heterogeneous mixing, limiting the ability to detect.

Finally, our results indicated that one-third of reviewed articles primarily focused on describing related to monitoring severity, and risk factors for severe disease, and assessing the impact on healthcare systems of swine influenza (ie, ‘objective 2’). Objective 2 entails the systematic observation of the severity of swine influenza cases, identifying risk factors associated with severe illness, and most studies reviewed used a combination of nasal samples with additional organ samples, which are adequate to evaluate viral pathogenesis. Additionally, it involves assessing the impact of swine influenza outbreaks on healthcare systems, including resource utilisation, strain on medical facilities and healthcare worker capacity. By closely monitoring severity and risk factors, stakeholders can better understand the demographic and health characteristics that contribute to severe cases, informing targeted interventions and resource allocation strategies. In addition, context-specific risk factors could be identified by local experts to inform the risk assessment to prioritise the interfaces or set for targeting surveillance. Although none of the articles specifically addressed ‘objective 4’, it is important to note that the efficacy of IAV vaccines is primarily evaluated in controlled experimental settings, which may not fully account for the complexities of real-world conditions. For instance, while seasonal influenza vaccines in humans exhibit varying effectiveness depending on the circulating strain, IAV vaccines for animals like swine are often tested in controlled environments that may not mirror the diverse conditions found on farms. Factors such as herd immunity, farm biosecurity practices and environmental variables can significantly influence vaccine coverage and effectiveness, highlighting the need for broader field-based studies. There are a few limitations that need to be mentioned when interpreting the results of the present study. Most of the published literature describes surveillance systems in project mode with limited information on routine systems for surveillance of swine influenza viruses. The quality of the included studies could not be assessed for risk of bias due to the diverse nature of individual study designs.

## Conclusion

Strengthening surveillance for IAV, particularly at the swine-human interface, is an important pandemic prevention and global health security strategy. Individual member states should take up exercises to document existing human-swine interfaces and estimate risk levels for the emergence of zoonotic IAVs and their spillage to humans. Based on the identified risk level, appropriate surveillance strategies may be implemented, considering the feasibility and economic viability. This scoping review has formed the basis for the next steps for the research team, which is, with the support of regional experts, to further map unpublished initiatives for surveillance of IAVs at the swine-human interface in the region, draw lessons from these initiatives and assess the risks and the need for establishing surveillance globally including SEAR. Following this, the research team proposes to develop operational surveillance guidance for national programme managers for systematically assessing the need and establishing a context-specific surveillance system for IAVs on the swine-human interface in SEAR member states. Through a process of extensive expert consultation and validation, the guidance is proposed to be finalised before being offered for rollout in the member states of WHO’s South East Asian region.

## Supplementary material

10.1136/bmjph-2024-002330 online supplemental table 1

10.1136/bmjph-2024-002330 online supplemental table 2

## Data Availability

Data are available upon reasonable request. All data relevant to the study are included in the article or uploaded as supplementary information.
