# Supplementary material for: Implications for influenza A virus surveillance in Southeast Asian Region countries: a scoping review of approaches for the surveillance of swine influenza viruses at human-swine interfaces
Source: BMJ Public Health. 2025 Jun 18;3(1):e002330. doi: 10.1136/bmjph-2024-002330 (PMC12182126; doi:10.1136/bmjph-2024-002330)
Supplement: online supplemental table 1 [file bmjph-3-1-s001.docx]

# Supplementary Table S1: Search Strategy

## A comprehensive overview of the search strategies, including the databases used, keywords, and search parameters for PubMed, Scopus and Web of Science, were provided in the tables below:

## Table A: PubMed (Title/Abstract)

| **Item** | **(PubMed)** | **No. of Articles** |
| --- | --- | --- |
| #1 Surveillance | (Surveillance[Title/Abstract]) OR (public health surveillance[MeSH Terms]) OR (monitoring[Title/Abstract]) | 7,46,674 |
| #2 Influenza | (Influenza zoonosis*[Title/Abstract]) OR (zoonotic Influenza[Title/Abstract]) OR (Swine influenza[Title/Abstract]) OR Influenza A[Title/Abstract] OR SIV[Title/Abstract] OR IAV[Title/Abstract] OR Influenza virus*[Title/Abstract] OR IAVs[Title/Abstract] | 65,662 |
| #3 Human | (Humans [Mesh]) OR (human*[Title/Abstract]) OR (Pig Farmer*[Title/Abstract]) OR (swine farmer*[Title/Abstract]) OR (pork farmer*[Title/Abstract]) OR (pig trader*[Title/Abstract]) OR (swine trader*[Title/Abstract]) OR (live market residents[Title/Abstract]) OR (pig handle*[Title/Abstract]) OR (Swine handle*[Title/Abstract]) OR (pig farm worker*[Title/Abstract]) OR (slaughter house worker*[Title/Abstract]) OR (swine worker*[Title/Abstract]) OR (pig worker*[Title/Abstract]) OR (swine farm worker*[ Title/Abstract]) OR (pig seller*[ Title/Abstract]) OR (swine seller*[ Title/Abstract]) OR (pork seller*[ Title/Abstract]) | 20,560,924 |
|  |  |  |
| #4 Swine | (Pig [Title/Abstract]) OR (Swine* [Mesh]) OR (Hog [Title/Abstract]) OR (Boar [Title/Abstract]) OR (Warthogs [Title/Abstract]) | 316,379 |
| #5 Spillover | (Spill over [Title/Abstract]) OR (Spillover [Title/Abstract]) OR (Transmission [Title/Abstract]) OR (Spread [Title/Abstract]) OR (Transmissibility [Title/Abstract]) OR (Spillage [Title/Abstract]) | 591,018 |
|  |  |  |
| #6 Interface | (pig Abattoir*[Title/Abstract]) OR (Pork Abattoir*[Title/Abstract]) OR (Swine Abattoir*[Title/Abstract]) OR (Swine market*[Title/Abstract]) OR (Animal market*[Title/Abstract]) OR (Pig production[Title/Abstract]) OR (Swine production[Title/Abstract]) OR (Swine trading[Title/Abstract]) OR (Pig trading[Title/Abstract]) OR (Pork trading[Title/Abstract]) OR (Pig slaughterhouse[Title/Abstract]) OR (Pork slaughterhouse[Title/Abstract]) OR (Swine slaughterhouse[Title/Abstract]) OR (interface[Title/Abstract]) OR (interaction[Title/Abstract]) OR (linkage[Title/Abstract]) OR (Mixing[Title/Abstract]) OR (landscape[Title/Abstract]) OR (Pig market[Title/Abstract]) OR (pork market[Title/Abstract]) OR (swine exposure[Title/Abstract]) OR (farm*[Title/Abstract]) OR (farming[Title/Abstract]) | 1,351,002 |
| #7 | #1 AND #2 | 6,077 |
| #8 | #3 OR #4 | 20,792,088 |
| #9 | #5 OR #6 | 1,894,436 |
| #10 | (#7) AND (#8) AND (#9) | 1,517 |

## Table B: Scopus (Title OR Abstract)

| **Item** | **(Scopus)** | **No. of Articles** |
| --- | --- | --- |
| #1 Surveillance | (Surveillance) OR (public health surveillance) OR (monitoring) | 2,928,779 |
| #2 Influenza | (Influenza zoonosis*) OR (zoonotic Influenza) OR (Swine influenza) OR Influenza A OR SIV OR IAV OR Influenza virus* OR IAVs | 133,958 |
| #3 Human | (Humans) OR (human*) OR (Pig Farmer*) OR (swine farmer*) OR (pork farmer*) OR (pig trader*) OR (swine trader*) OR (live market residents) OR (pig handle*) OR (Swine handle*) OR (pig farm worker*) OR (slaughter house worker*) OR (swine worker*) OR (pig worker*) OR (swine farm worker*) OR (pig seller*) OR (swine seller*) OR (pork seller*) | 28,281,595 |
|  |  |  |
| #4 Swine | (Pig) OR (Swine*) OR (Hog) OR (Boar) OR (Warthogs) | 570,079 |
| #5 Spillover | (Spill over) OR (Spillover) OR (Transmission) OR (Spread) OR (Transmissibility) OR (Spillage) | 3,112,925 |
|  |  |  |
| #6 Interface | (pig Abattoir*) OR (Pork Abattoir*) OR (Swine Abattoir*) OR (Swine market*) OR (Animal market*) OR (Pig production) OR (Swine production) OR (Swine trading) OR (Pig trading) OR (Pork trading) OR (Pig slaughterhouse) OR (Pork slaughterhouse) OR (Swine slaughterhouse) OR (interface) OR (interaction) OR (linkage) OR (Mixing) OR (landscape) OR (Pig market) OR (pork market) OR (swine exposure) OR (farm*) OR (farming) | 8,679,891 |
| #7 | #1 AND #2 | 15,327 |
| #8 | #3 OR #4 | 156,786 |
| #9 | #5 OR #6 | 379,089 |
| #10 | (#7) AND (#8) AND (#9) | 298 |

## Table C: Web of Science (Title OR Abstract)

| **Item** | **Web of Science** | **No. of Articles** |
| --- | --- | --- |
| #1 Surveillance | (Surveillance) OR (public health surveillance) OR (monitoring) | 2,052,205 |
| #2 Influenza | (Influenza zoonosis*) OR (zoonotic Influenza) OR (Swine influenza) OR Influenza A OR SIV OR IAV OR Influenza virus* OR IAVs | 97,313 |
| #3 Human | (Humans) OR (human*) OR (Pig Farmer*) OR (swine farmer*) OR (pork farmer*) OR (pig trader*) OR (swine trader*) OR (live market residents) OR (pig handle*) OR (Swine handle*) OR (pig farm worker*) OR (slaughter house worker*) OR (swine worker*) OR (pig worker*) OR (swine farm worker*) OR (pig seller*) OR (swine seller*) OR (pork seller*) | 3,816,192 |
|  |  |  |
| #4 Swine | (Pig) OR (Swine*) OR (Hog) OR (Boar) OR (Warthogs) | 192,547 |
| #5 Spillover | (Spill over) OR (Spillover) OR (Transmission) OR (Spread) OR (Transmissibility) OR (Spillage) | 1,692,355 |
|  |  |  |
| #6 Interface | (pig Abattoir*) OR (Pork Abattoir*) OR (Swine Abattoir*) OR (Swine market*) OR (Animal market*) OR (Pig production) OR (Swine production) OR (Swine trading) OR (Pig trading) OR (Pork trading) OR (Pig slaughterhouse) OR (Pork slaughterhouse) OR (Swine slaughterhouse) OR (interface) OR (interaction) OR (linkage) OR (Mixing) OR (landscape) OR (Pig market) OR (pork market) OR (swine exposure) OR (farm*) OR (farming) | 6,013,528 |
| #7 | #1 AND #2 | 12,493 |
| #8 | #3 OR #4 | 41,097 |
| #9 | #5 OR #6 | 237,029 |
| #10 | (#7) AND (#8) AND (#9) | 178 |
