# Supplementary material for: Implications for influenza A virus surveillance in Southeast Asian Region countries: a scoping review of approaches for the surveillance of swine influenza viruses at human-swine interfaces
Source: BMJ Public Health. 2025 Jun 18;3(1):e002330. doi: 10.1136/bmjph-2024-002330 (PMC12182126; doi:10.1136/bmjph-2024-002330)
Supplement: online supplemental table 2 [file bmjph-3-1-s002.docx]

**Supplementary Table S2**: The details of relevant information extracted from selected articles were summarised in the table below:

| SL.No | **Study ID/Ref.** | **Target species (Human/pigs/others)** | **Types of Surveillance** | **Objective 1** | **Objective 2** | **Objective 3** | **Objective 4** | **Period/ Duration** | **Country** | **Surveillance setting** | **Type of Samples collected** | **Laboratory Methods** |
| --- | --- | --- | --- | --- | --- | --- | --- | --- | --- | --- | --- | --- |
| 1 | Henritzi et al 2020 | Swine | Passive | Yes | Yes | Yes | No | 2015 - 2018 | 17 European countries | Pig farms | Nasal swabs | PCR and Whole-genome Sequencing |
| 2 | Hervé S et al 2019 | Swine | Passive | Yes | Yes | Yes | No | 2011 - 2018 | France | Pig farms | Nasal swabs | PCR |
| 3 | Hansen_2021 | Swine | Passive | Yes | No | Yes | No | 2011 - 2018 | Denmark | Pig farms | Nasal swabs and Lung tissues | PCR and Whole-genome Sequencing |
| 4 | Abe et al., 2015 | Swine | Active | Yes | No | Yes | No | 2011-2014 | Thailand | Pig farms | Nasal swabs | PCR and Whole-genome Sequencing |
| 5 | Bowman et al., 2012 | Swine | Active | Yes | No | Yes | No | 2009 - 2011 | USA | Agricultural fairs | Nasal swabs | PCR |
| 6 | Cao et al., 2019 | Swine | Active | No | No | Yes | No | 2013 - 2015 | China | Pig farms and slaughterhouses | Nasal swabs | PCR and Whole-genome Sequencing |
| 7 | Charoenvisal et al., 2013 | Swine | Active | No | No | Yes | No | 2010 - 2012 | Thailand | Pig farms | Nasal swabs and Lung tissues | PCR and Whole-genome Sequencing |
| 8 | Jimenez-Bluhm et al 2018 | Swine, Poultry | Active | Yes | No | Yes | No | 2012 - 2014 | Chile | Backyard Productive Systems (BPS) | Cloacal and tracheal swabs and Serum samples | PCR and Whole-genome Sequencing |
| 9 | Mine-2020 | Swine | Active | Yes | No | Yes | No | 2015 - 2019 | Japan | Pig farms | Nasal swabs and Lung tissues | PCR and Whole-genome Sequencing |
| 10 | Russell_2009 | Swine, human | Active | No | No | Yes | No | 2008 | Canada | Pig farms | Nasal and tracheal swabs, serum samples | PCR |
| 11 | Terebuh_2010 | Swine, human | Active | Yes | Yes | Yes | No | 2002 - 2004 | USA | Pig farms | Nasal swabs and serum samples | PCR |
| 12 | USDA's IAV-S | Swine | Active | Yes | Yes | Yes | No | 2010 - ongoing | USA | Pig farms | Lung, nasal swab and oral fluids | PCR and Whole-genome Sequencing |
| 13 | Yin_2014 | Farm Worker | Active | Yes | No | No | No | 2011 - 2012 | China | Pig farms | Serum samples | ELISA |
| 14 | Yang_2016 | Swine | Active | No | No | Yes | No | 2010 - 2013 | China | Slaughterhouses | Nasal swabs | Whole-genome sequencing |
| 15 | Zhu_2011 | Swine | Active | Yes | No | Yes | No | 2009 - 2010 | China | slaughterhouses | Nasal and tracheal swabs, serum samples | ELISA |
| 16 | Influenza Surveillance Program in Pigs, Hong Kong | Swine | Active | Yes | Yes | No | No | 2012 - 2015 | Hong Kong | Slaughterhouses | Nasal and tracheal swabs, serum samples | PCR |
| 17 | Kaplan_2015 | Swine | Active | Yes | Yes | Yes | No | 2012 – 2013 | USA | Pig farms | Nasal swabs | PCR |
| 18 | Sun_2020 | Swine, human | Active | No | No | Yes | No | 2011 - 2018 | China | Pig farms | Nasal swabs and Lung tissues | PCR and Whole-genome Sequencing |
| 19 | Takemae_2017 | Swine | Active | No | No | Yes | No | 2010 - 2015 | Vietnam | Pig farms and slaughterhouses | Nasal swabs | Whole-genome sequencing |
| 20 | Wu_2011 | Swine | Active | Yes | No | Yes | No | 2008 - 2009 | China | Pig farms | Nasal swabs and serum samples | PCR and Whole-genome Sequencing |
| 21 | Anderson et al., 2018 | Swine, human, environment | Active | Yes | Yes | No | No | 2015 - 2019 | China | Pig farms | Nasal swabs, fecal, and aerosol samples | PCR and Whole-genome Sequencing |
| 22 | Anderson et al., 2020 | Swine | Active | Yes | Yes | No | No | 2017 - 2017 | USA | Pig farms | Nasal swabs, fecal, and aerosol samples | PCR |
| 23 | Baudon et al, 2020 | Swine | Active | Yes | Yes | No | No | 2013 - 2014 | Vietnam | Pig farms | Nasal swabs and serum samples | ELISA and Whole-genome sequencing |
| 24 | Choi et al., 2015 | Swine, human, environment | Active | Yes | Yes | No | No | 2012 - 2013 | USA | Animal markets | Nasal swabs, fecal, and aerosol samples | PCR and Whole-genome Sequencing |
| 25 | Choi et al., 2014 | Live Animal Market workers | Active | No | Yes | No | No | 2008 - 2012 | USA | Animal markets | Nasal swabs | PCR and Whole-genome Sequencing |
| 26 | Corzo et al., 2013 | Swine | Active | No | Yes | No | No | 2009 - 2011 | USA | Pig farms | Nasal swabs | PCR |
| 27 | Diaz et al., 2015 | Swine | Active | Yes | Yes | No | No | 2011 - 2012 | USA | Pig farms | Nasal swabs | PCR |
| 28 | Er C et al 2016 | Swine | Active | Yes | Yes | No | No | 2010 - 2014 | Norway | Pig farms and slaughterhouses | Nasal swabs | PCR |
| 29 | He P et al., 2018 | Swine | Active | No | No | Yes | No | 2013 - 2015 | China | Pig farms and slaughterhouses | Nasal and tracheal swabs, serum samples | PCR and Whole-genome Sequencing |
| 30 | Lekcharoensuk_2010 | Swine | Active | No | No | Yes | No | 2004 - 2006 | Thailand | Pig farms | Nasal swabs and Lung tissues | PCR and Whole-genome Sequencing |
| 31 | Ma MJ_2018 | Swine, human, environment | Active | No | No | Yes | No | 2015 - 2019 | China | Pig farms | Nasal swabs, fecal, and aerosol samples | PCR and Whole-genome Sequencing |
| 32 | Meseko_2014 | Swine, human | Active | Yes | No | No | No | 2010 - 2012 | Nigeria | Pig farms | Nasal swabs | PCR |
| 33 | Mine-2019 | Swine | Active | No | No | Yes | No | 2011 - 2017 | Thailand | Pig farms | Nasal swabs | PCR and Whole-genome Sequencing |
| 34 | Mon_2020 | Swine | Active | No | No | Yes | No | 2017 – 2019 | Myanmar | Pig farms | Nasal swabs | PCR and Whole-genome Sequencing |
| 35 | Nirmala _2020 | Swine | Active | No | No | Yes | No | 2012 - 2013 | USA | Animal markets | Lung tissue samples | PCR |
| 36 | Nonthabenjawan_2015 | swine | Active | No | No | Yes | No | 2011 - 2014 | Thailand | Pig farms | Nasal swabs | PCR and Whole-genome Sequencing |
| 37 | Sobolev _2019 | Swine | Active | No | No | Yes | No | 2016 - 2017 | Russia | Pig farms | Lung tissue samples | Whole-genome sequencing |
| 38 | Song_2010 | Swine | Active | Yes | No | No | No | 2004 & 2007 | China | Pig farms | Serum samples | ELISA |
| 39 | Vijaykrishna _2011 | Swine | Active | Yes | No | Yes | No | 1998 - 2010 | China | Slaughterhouses | Nasal and tracheal swabs, serum samples | Whole-genome sequencing |
| 40 | Van Reeth_2008 ESNIP | Swine | Active | Yes | No | No | No | 2001– 2004 | 13 European Countries | Pig farms | Serum samples | ELISA |
| 41 | Nelson_2020 | Swine | Joined | Yes | No | Yes | No | 2010 | USA | Agricultural fairs | Nasal swabs | PCR and Whole-genome Sequencing |
| 42 | Szablewski_2023 | Swine, human | Active | Yes | No | Yes | No | 2013-2015 | USA | Agricultural fairs | Nasal swabs | PCR and Whole-genome Sequencing |
